# Viscera Characteristics of *MSTN*-Edited Heterozygous Pigs

**DOI:** 10.3389/fgene.2022.764965

**Published:** 2022-03-01

**Authors:** Yangli Pei, Ziyao Fan, Yuxin Song, Chujie Chen, Yulian Mu, Bugao Li, Zheng Feng, Hua Li, Kui Li

**Affiliations:** ^1^ Guangdong Provincial Key Laboratory of Animal Molecular Design and Precise Breeding, Key Laboratory of Animal Molecular Design and Precise Breeding of Guangdong Higher Education Institutes, School of Life Science and Engineering, Foshan University, Foshan, China; ^2^ Institute of Animal Sciences, Chinese Academy of Agricultural Sciences, Beijing, China; ^3^ Genome Analysis Laboratory of the Ministry of Agriculture, Agricultural Genomics Institute at Shenzhen, Chinese Academy of Agricultural Sciences, Shenzhen, China; ^4^ College of Animal Science, Shanxi Agricultural University, Taigu, China; ^5^ Shenzhen Branch, Guangdong Laboratory of Lingnan Modern Agriculture, Genome Analysis Laboratory of the Ministry of Agriculture and Rural Affairs, Agricultural Genomics Institute at Shenzhen, Chinese Academy of Agricultural Sciences, Shenzhen, China

**Keywords:** myostatin, pigs, heterozygote, visceral, histomorphology

## Abstract

Myostatin (MSTN) is a protein that negatively regulates growth of skeletal muscle, and inactivation of MSTN improves the mass of skeletal muscle. Our previous work found that *MSTN* ^*+/−*^ pigs have higher muscle depth and lower fat depth compared to wild type without any developmental problems. Therefore, *MSTN*-edited pigs are most likely to appear as heterozygotes in the potential future market, but the characteristics of organs in digestive and reproductive system of pigs with *MSTN* gene editing remains unclear. Here, we investigated the histological of the organs in the digestive system and reproductive system in *MSTN* gene heterozygotes at adult stages. The length of intestine was further compared between adult heterozygous and wild type pigs. We found no significant differences in histomorphology of organs, including heart, duodenum, jejunum, ileum, cecum, colon, testis, epididymis, ovaries, oviducts and uterus, between individuals from two genotypes. Moreover, there was no significant difference in the average length of intestine in adult pigs. Our data provide a reference for further clarifying the applications of *MSTN* gene edited pigs.

## Introduction

In 1997, McPherron first identified MSTN (also known as growth differentiation factor-8) as a member of the TGF-β (transforming growth factor-β) superfamily (McPherron et al., 1997). *MSTN* is highly expressed in skeletal muscle, but lowly expressed in adipose tissue and cardiac muscle (McPherron et al., 1997; Sharma et al., 1999). MSTN strongly affects skeletal muscle development (McPherron and Lee, 1997; Zhao et al., 2005; Guo et al., 2009; Ahad et al., 2017). The *MSTN* mutation lead to a double muscling phenotype in cattle (Grobet et al., 1997; McPherron and Lee, 1997), mice (Bünger et al., 2004), sheep (Clop et al., 2006) and other animals (Schuelke et al., 2004; Mosher et al., 2007).

Since pigs are farmed as a major source of animal protein for humans, generation of pigs with *MSTN* loss-of-function mutations has become a research priority in order to obtain breeding stock with better meat quality and higher economic value. Previous studies have reported the successful production of healthy *MSTN* gene-inactivated Chinese Meishan pigs (Qian et al., 2015) and Erhualian pigs (Wang et al., 2017). In addition to phenotypic changes to muscle and fat tissues, organs weights were generally significantly reduced among *MSTN* ^*−/−*^ animals compared with the organs in their wild type (WT) counterparts (Bünger et al., 2004; Fiems, 2012; Luo et al., 2019). Bünger (2004) found that in MSTN knockout mice, the significant increase in muscle weight and decrease in body fat content were accompanied by significant reductions in the weights of heart, liver, kidney and digestive tract (Bünger et al., 2004). Luo (2019) examined the organ weights of *MSTN* ^*−/−*^, *MSTN* ^*+/−*^ and WT piglets and found that the visceral weight of *MSTN* ^*−/−*^ homozygous piglets was significantly lower than that of *MSTN* ^*+/−*^ heterozygotes and WT piglets (Luo et al., 2019). These studies show that animals with double site knockout of *MSTN* have lighter internal organs than WT individuals. The development of organs has a direct impact on an individual’s health, and the weights of body tissues and organs directly affect the slaughter rate, an important index of pig slaughtering performance, which in turn affects economic benefits. However, the mechanism of how MSTN regulates visceral development remains unclear. These findings all raise concerns about the health and welfare of *MSTN* edited animals.

In our previous study, *MSTN* ^*+/−*^ Large White (LW) pigs underwent exon 3 editing were prepared. The lean meat production of the *MSTN* ^*+/−*^ LW pigs were significantly higher than that of the WT (75.50%: 69.44%, *p* < 0.0001). Conversely, the fat meat rates of *MSTN* ^*+/−*^ pigs were significantly lower than that of the WT pigs (5.11%: 10.36%, *p* = 0.0022). The majority of amino acids content in the *MSTN*-edited LW pork were significantly higher than that in the WT pork; and the levels of polyunsaturated fatty acids were enriched in the *MSTN*-edited LW lean meat (Fan et al., 2021). Pigs with a naturally occurring single copy *MSTN* mutation have higher muscle depth and lower fat depth compared to the WT (Matika et al., 2019). Therefore, the heterozygous of *MSTN* gene-edited pigs have excellent production performance and are likely to be used in market applications in the future. However, there has been no systematic study on the digestive tract organs and reproductive organs of *MSTN* ^*+/−*^ pigs. The structure and function of intestine can affect nutrient absorption from feed. And the function of reproductive system organs determines the reproductive efficiency of the breeding herd. These are important factors to the economic benefit of pig production. Therefore, we examined the histological features of major organs in digestive and reproductive system in adults, and also contrasted the intestine length of *MSTN* ^*+/−*^ LW pigs and their WT half-sibs. This work will provide an important reference for *MSTN* gene edited pigs that can be used in future pig breeding.

## Material and Methods

### Animals

The pigs had *ad libitum* access to a commercial pig diet and water throughout the experimental period. All animal studies were approved by the Animal Welfare and Research Ethics Committee at the Institute of Animal Sciences, Chinese Academy of Agricultural Sciences. Here, we used newborn, 5-month-old and 8-month-old *MSTN* ^*+/−*^ and WT pigs to study the viscera development characteristics.

### Histological Analysis

Paraffin-embedded sections (4 μm thick) of various organs of *MSTN* ^*+/−*^, and WT piglets, 5-month-old pigs, and 8-month-old pigs were made using a histotome. The slices were dewaxed, hydrated with gradient alcohol, stained with hematoxylin solution for 15 min, counterstained with 0.5% eosin solution for 5 min, dehydrated with gradient alcohol, cleared, and sealed (Feldman and Wolfe, 2014). After staining, images were acquired using a LEICA DMi8 microscope. Then, we assessed which cell types were in the images and the morphological characteristics of the cells to detect whether there were any changes in the tissue structure between the two genotypes.

### Characterization and Analysis of Organs

In total, 17 *MSTN* ^*+/−*^ and 18 WT LW pigs were slaughtered at 8 months old, and the length of intestinal length was measured as follows:

Small intestinal length (m): Length from the pylorus to the ileocecal junction.

Large intestine length (m): The length from the ileocecal junction to the anus.

### Statistical Analysis

Data were analyzed using SPSS 19 software (SPSS, Inc., Chicago, IL, United States). All data were expressed as Mean ± SEM. The independent sample *t*-test (Student’s *t*-test) was used to calculate the significance, and *p* < 0.05 was used as the criterion for significance.

## Results

### No Significant Changes in the Histomorphology of Heart Between WT and *MSTN* ^*+/−*^ Pigs

**FIGURE 1:**
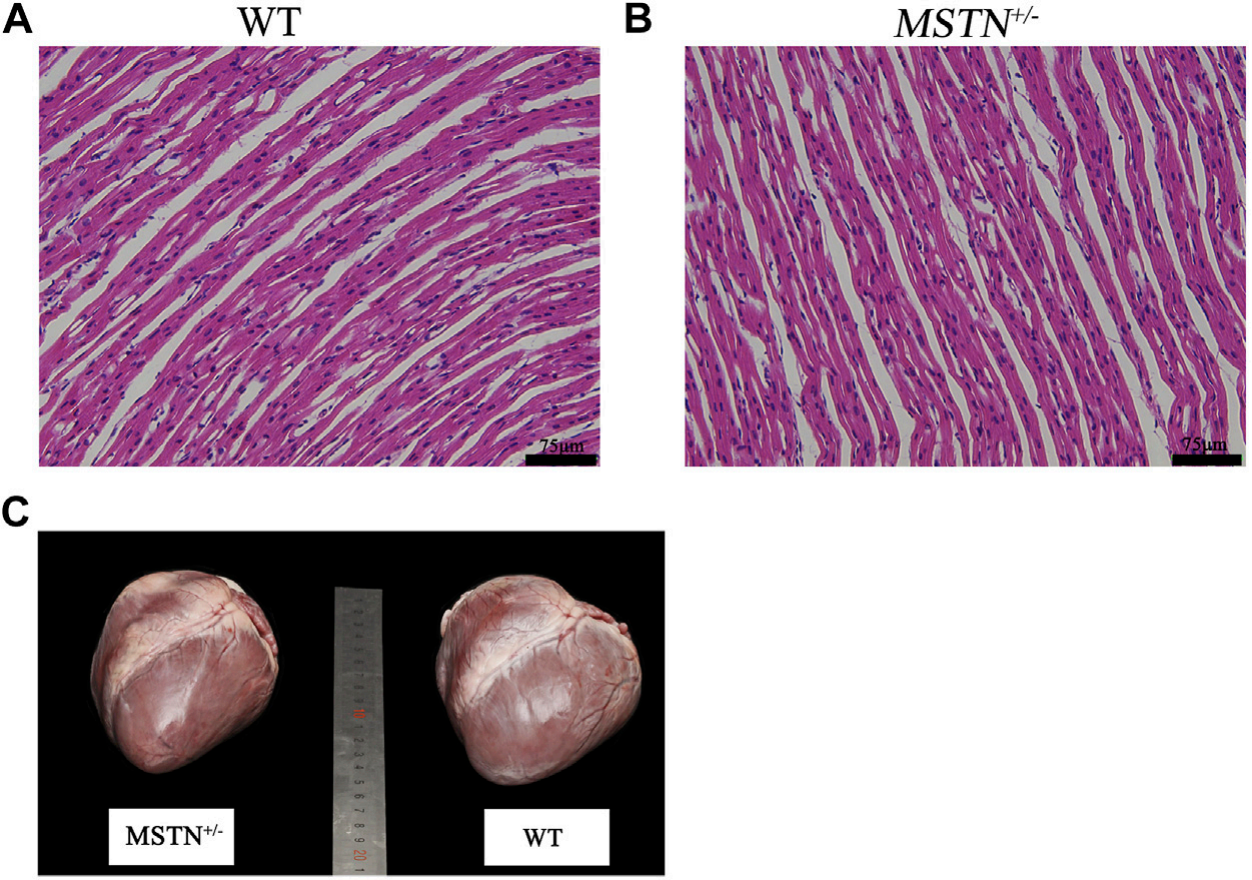
Morphological analysis of heart in *MSTN* ^+/−^ and WT pigs. **(A)** *HE* staining results of heart tissue in WT piglets. **(B)** *HE* staining results of heart tissue in *MSTN* ^+/−^ piglets. **(C)** Morphology of heart in 8-month-old *MSTN* ^+/−^ and WT pigs.

The formalin-fixed paraffin-embedded heart tissue of *MSTN* ^*+/−*^ and WT piglets were stained by Hematoxylin and Eosin (*H*&*E*) for histomorphological comparison. Results showed that the myocardial fibers of newborn WT and MSTN pigs were both short and cylindrical, branched and interconnected in a network, and there were intercalated discs at the junction between adjacent myocardial fibers (Figures 1A,B). Furthermore, we compared the sizes and weights of heart of 17 *MSTN* ^*+/−*^ and 18 WT 8-month-old pigs. The appearance and sizes of heart did not markedly differ between the *MSTN* ^*+/−*^ and WT adult pigs (Figure 1C). There was also, no significant difference of average heart weight between *MSTN* ^*+/−*^(406.5 ± 15 g) and WT (452.3 ± 32.4 g).

### No Significant Differences in Histomorphology of Major Organs in Digestive System Between Individuals From Two Genotypes at 5-month-Old

**FIGURE 2:**
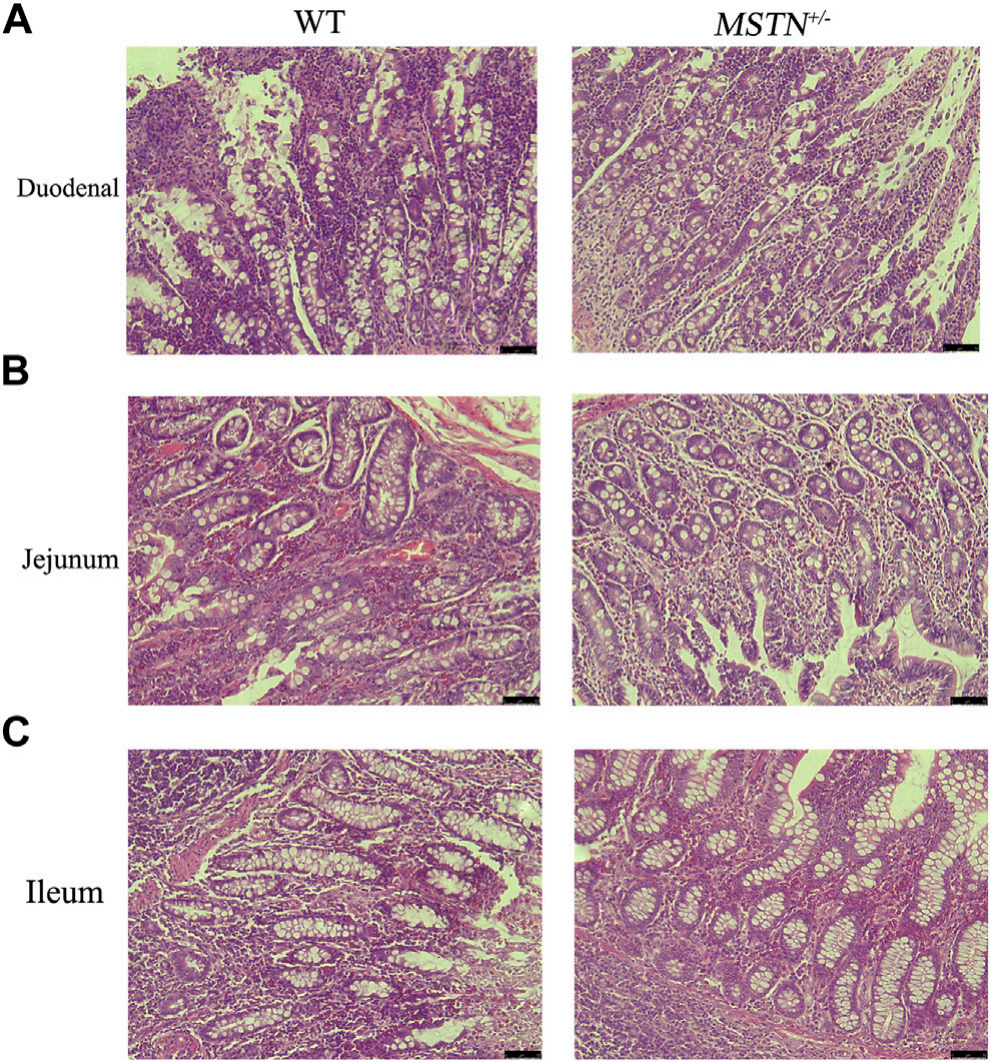
Histological analysis of duodenum, jejunum, and ileum in 5-month-old *MSTN* ^+/−^ and WT pigs. **(A–C)** Representative pictures of *HE* staining in duodenum **(A)**, jejunum **(B)**, and ileum tissue of WT (left) and *MSTN* ^+/−^ (right) pigs.

**FIGURE 3:**
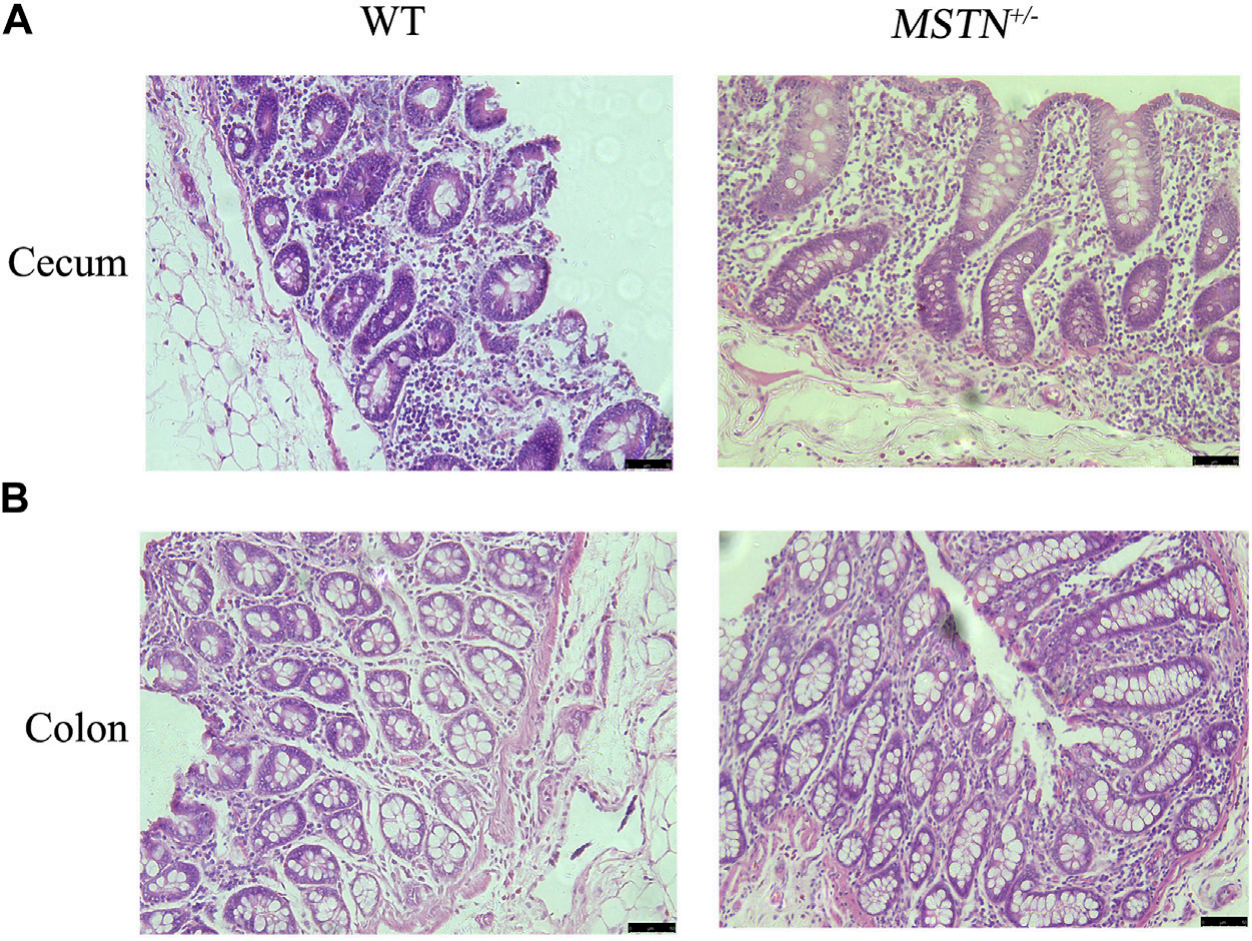
Histological analysis of cecum and colon in 5-month-old *MSTN* ^+/−^ and WT pigs. **(A,B)** Histology of cecum **(A)** and colon **(B)** from WT (left) and *MSTN* ^+/−^ (right) pigs was investigated by *HE* staining.

When pigs were 5 months old, we carried out histomorphological examination of the duodenum, jejunum, ileum, cecum, and colon. The structure of duodenum, jejunum and ileum of pigs was intact, and the small intestinal villi were slender and orderly arranged, the morphology and structure of epithelial cells were intact and clear, the cup cells were evenly distributed and in large number, the villi height was normal, the morphology and structure of crypt were clear, and the depth of crypt was normal (Figures 2A–C). The colonic structure was intact. The epithelial cells of the mucosa are arranged neatly and clearly defined, and the boundary between the epithelial layer and lamina propria is clear. The columnar cells are tall and the nuclei are elliptic, and the goblet cells are goblet shaped near the base of the cells (Figure 3A). The structure of the cecum is complete and closely arranged as that of WT pigs. The villi are orderly, uniform in length and very dense (Figure 3B). Furthermore, we compared the intestinal length of 17 *MSTN* ^*+/−*^ and 18 WT 8-month-old pigs. No any significant difference between genotypes in total intestinal length (combined length of small and large intestines, Table 1). According to the results, there were no significant morphological and length differences between *MSTN* ^+/−^ and WT in these tissues.

**TABLE 1:** The intestinal length of 8-month-old *MSTN* ^+/−^ and WT pigs.

Measure	*MSTN* ^+/−^	WT
Total intestinal length (m)	25.2288 ± 0.5191	25.7839 ± 0.4631
Length of small intestine (m)	20.3200 ± 0.3069	20.5700 ± 0.4047
Length of large intestine (m)	5.2075 ± 0.1594	5.2139 ± 0.1097
Length of small intestine/BL (m/cm)	0.2031 ± 0.0032	0.2067 ± 0.0036
Length of large intestine/BL (m/cm)	0.0518 ± 0.0015	0.0524 ± 0.0011
Total intestinal length/BL (m/cm)	0.2518 ± 0.0049	0.2591 ± 0.0041

All values are presented as Mean ± SEM.

### No Significant Changes in the Reproductive Organs Between Adult WT and *MSTN* ^*+/−*^ Pigs at 8-month-Old

**FIGURE 4:**
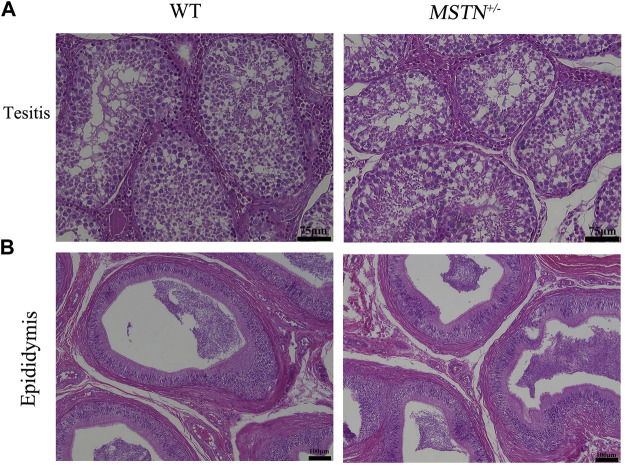
Histological of testis and epididymitis in 8-month-old *MSTN* ^+/−^ and WT pigs. **(A,B)** *HE* staining of testicular **(A)** and epididymal **(B)** tissue in WT (left) and *MSTN* ^+/−^ (right) pigs.

**FIGURE 5:**
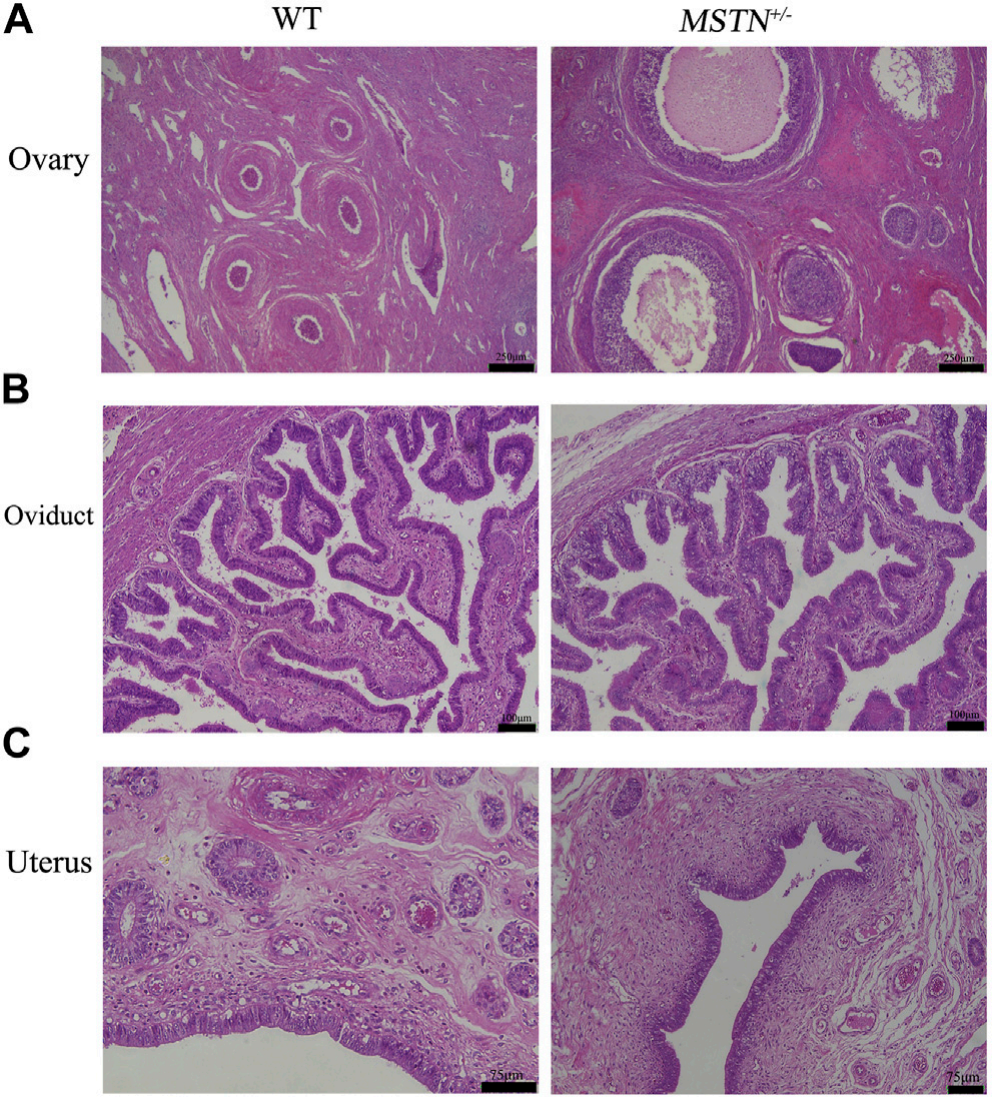
Histological analysis of ovary, oviduct and uterus in 8-month-old *MSTN* ^+/−^ and WT sow. **(A–C)** Ovarian **(A)**, tubal **(B)**, and uterine **(C)** histology image from WT (left) and *MSTN* ^+/−^ (right) sow.

Reproductive organs, including testis, epididymis, ovaries, oviducts and uterus, were collected from pigs aged 8 months. And these tissues were processed for *H&E* staining. The *MSTN* ^+/−^ pigs showed normal histology of testis and epididymides, and sperm were present in testis and epididymides (Figure 4). Different follicular stages and atretic follicles existed in ovarian sections (Figure 5A). Compared with the WT group, histological analysis under light microscopy showed no morphologic differences in the ovaries, oviducts, and uterus histology of the MSTN group (Figure 5).

## Discussion

Many laboratories have encountered problems at different stages in the process of *MSTN* deletion or editing. For example, *MSTN*-knockout Large White/Landrace×Duroc piglets died within 24 h after birth, and weighed less at birth than WT piglets. The single surviving piglet was sacrificed at 29 days old because of weakness in its hindlimbs (Rao et al., 2016). Similarly, eight of the *MSTN* ^*−/−*^ Landrace newborns generated with CRISPR/Cas9 editing died within a week (Wang et al., 2015). Pigs with naturally occurring MSTN mutations were reported in 2019 (Matika et al., 2019). However, piglets that were homozygous for this mutant allele suffered from lameness syndrome and did not survive when growth with the live weight above 40 kg (Matika et al., 2019). *MSTN* homozygous knockout in LW pigs leads to lameness, in agreement with other studies. Subsequently, pigs used for this study carrying single, edited copies of *MSTN* were propagated to expand the population for up to 8 years, which was sufficient to show that these *MSTN* ^*+/−*^ swine exhibited no developmental defects related to the mutant allele. *MSTN* ^*+/−*^ heterozygotes had a significant decrease in fat content that was accompanied by an increase in lean meat content (Fan et al., 2021). Therefore, *MSTN*-edited pigs are most likely to appear as heterozygotes in the potential future market.

Several internal organs have a reduced size in *MSTN* ^*−/−*^ homozygous mutant animals compared with WT (Bünger et al., 2004; Fiems, 2012; Luo et al., 2019). The Belgian Blue double-muscled (BBDM) calves (11 base-pare deletion of the MSTN (Kambadur et al., 1997)) had a reduction of 18% for the digestive tract, and 14% for the heart (Fiems, 2012). Another study reported that mice carrying homozygous mutations in murine MSTN had significantly lower weights (12–20%) for organs including liver, kidney, heart and digestive tract compared to homozygous WT mice (Bünger et al., 2004). Similarly, neonatal *MSTN* ^*−/−*^ piglets had decreased heart, liver, lungs, kidney, and stomach weights relative to body weight by 21.4, 21.3, 29.8, 16.7, and 20.0%, respectively (Luo et al., 2019).

Cardiac performance, reserve, and capability of the smaller heart are reduced in *MSTN* ^*−/−*^ animals compared to WT (Amory et al., 1993). The lower cardiac performance of *MSTN* ^*−/−*^ animals leading more quickly to exhaustion after severe exercise (Monin et al., 1974), which may even terminate in sudden death (Holmes et al., 1973). Our results showed that there were no histological abnormalities of cardiac muscle structures, and no significant difference in weight between individuals from two genotypes adults. *MSTN* ^*+/−*^ LW pigs did not suffer from reduced heart function, so there is no need to take extra precautions for animals.

It is well known that the small intestine consists of duodenum, jejunum, and ileum, is responsible for digestion and absorption of nutrients, water, and electrolytes, while the large intestine dividing into cecum and colon, is responsible to absorb water. Notably, the digestive disorders are related to intestinal dysfunction (Johnson, 1985; Soybel, 2005; Iji, 2008). Besides, the length of the digestive tract is positively correlated with nutrient absorption, while the decreases in the digestive tract length may limit the basic metabolism and nutrient availability of the body (Ortigues and Doreau, 1995; Greig et al., 2019). As reported by several authors, a consequence of the smaller digestive tract is reduced feed intake capacity (Clinquart et al., 1995; Fiems et al., 1997). The decrease in feed intake means that *MSTN* ^*−/−*^ animals do not efficiently utilize low quality feed, and the costs of rearing will increase. According to our results, the histological structure and length of intestinal in *MSTN* ^*+/−*^ swine were not affected by single copy *MSTN* mutation. This coincides with our previous finding that no differences were detected in feed conversion ratio values between *MSTN* ^*+/−*^ and WT LW (Fan et al., 2021). Hence, there were no differences between *MSTN* ^*+/−*^ and WT LW pigs in the function of digestive tract.

Spermatogenesis occurs in the testis and completes in the epididymis (de Kretser et al., 1998). Meanwhile, the ovary, oviduct and uterus are critical organs responsible for the oogenesis and embryonic development (Bagnell et al., 2017). The BBDM cows have significant longer calving interval than dairy cows (averaging 435 days compared to 393 days) (Hanzen et al., 1994). That may be caused by underdeveloped maternal reproductive tract and increased birth weight of calves, which make cesarean section an elective operation (Kolkman et al., 2007). A longer calving interval can be affected by the cesarean section, which significantly reduces the subsequent pregnancy rate (Patterson et al., 1981), but this is not the sole cause. Compared with Holstein cows, BBDM cows have significantly poorer semen quality (Hoflack et al., 2006). Nevertheless, BBDM cows have better egg quality than Holstein cows (Leroy et al., 2005). Other studies have shown that compared with the control group, MSTN-immunized mice had reduced litter number, but normal embryo development in both groups (Liang et al., 2007). These results are consistent with those obtained with BBDM (Leroy et al., 2005). This indicates that the completely loss of MSTN function does not affect the function of the uterus, but reduces the quality of sperm and increases litter birth weight. Our results previous work showed that the total number born, the number born alive and litter birth weight of the *MSTN* ^*+/−*^ piglets did not differ from those of the WT (Fan et al., 2021). Here, results showed that there was no significant difference in the histomorphology of testis, epididymis, ovaries, oviducts and uterus in *MSTN* ^*+/−*^ LW compared with WT pigs. Additionally, our *MSTN* ^*+/−*^ LW pigs are now in their eight generation (Fan et al., 2021). Therefore, it can be seen that the characteristics of reproductive organs of *MSTN* ^*+/−*^ LW pigs are similar to WT pigs.

## Conclusion

In summary, *MSTN* ^*+/−*^ LW pigs had no significant differences in the histomorphology of the heart, duodenum, jejunum, ileum, cecum, or colon, nor in the lengths of the large and small intestine compared to WT pigs. Similarly, no morphologic abnormality was evident in testis, epididymis, ovaries, oviducts and uterus. These results indicated that the characteristics of heart, digestive and reproductive organs of *MSTN* ^*+/−*^ heterozygotes are similar to that of WT LW pigs.

## Data Availability

The original contributions presented in the study are included in the article/Supplementary Material, further inquiries can be directed to the corresponding author.
